# Patterns and outcomes of immune-related adverse events in solid tumor patients treated with immune checkpoint inhibitors in Thailand: a multicenter analysis

**DOI:** 10.1186/s12885-021-09003-z

**Published:** 2021-11-25

**Authors:** Nuttapong Ngamphaiboon, Suthinee Ithimakin, Teerada Siripoon, Nattaya Sintawichai, Virote Sriuranpong

**Affiliations:** 1 Medical Oncology Unit, Department of Medicine, Faculty of Medicine, Ramathibodi Hospital, Mahidol University, Bangkok, Thailand; 2 Division of Medical Oncology, Department of Medicine, Faculty of Medicine Siriraj Hospital, Mahidol University, Bangkok, Thailand; 3 Division of Medical Oncology, Department of Internal Medicine, Faculty of Medicine, Chulalongkorn University & The King Chulalongkorn Memorial Hospital, 1873 Rama IV Rd., Pathumwan, Bangkok, 10330 Thailand

**Keywords:** cancer immunotherapy, Immune-checkpoint inhibitors, Immune-related adverse events, Overall survival, Thailand

## Abstract

**Background:**

Most immune-related adverse event (irAE) patterns and treatment guidelines are based on western clinical data. We evaluated the incidence and patterns of irAEs in patients treated with immune-checkpoint inhibitors (ICI) in Thailand.

**Methods:**

All solid tumor patients treated with ICIs were retrospectively reviewed in a multicenter analysis. The study aims to evaluate the incidence of irAEs and their characteristics, treatments, outcomes, and impact on survival. All irAEs were graded using the CTCAE version 4.0. Characteristics of irAEs including time to onset, duration of irAEs, specific treatments, and outcomes of irAEs were reviewed. The Chi-square or Fisher’s exact test was used to compare variables. Overall survival (OS) was estimated by the Kaplan-Meier method, and compared by the log-rank test. A *p*-value < 0.05 was considered statistically significant.

**Results:**

irAEs of any grade were observed in 98 of 414 patients (24%), whereas grades 3–4 irAEs were observed in 5.6%. The majority of patients (78%) were treated with monotherapy ICI (anti-PD1/PD-L1 92%). The most common all-grade irAEs were hypothyroidism (7.5%), hepatitis (6.5%), and rash (4.8%). Median onset of overall irAEs was 63 days. Pancreatitis and pneumonitis had the earliest onset at 30 and 34 days, respectively. ICIs were rechallenged in 68 of 98 patients with irAE. Eleven of sixty-eight patients (11.2%) with initial irAE had reoccurrence after ICI rechallenge. Based on a multivariate analysis, pre-existing hypothyroidism, ICI used in a clinical trial setting, and combinations of ICI/ICI were independent factors predicting irAE occurrence. Patients with irAE had a statistically significant longer overall survival (OS) when compared to patients without irAE (*p* = 0.019). A multivariate analysis revealed that occurrence of irAE was an independent prognostic factor for OS (HR 0.70, 95% CI 0.51–0.96; *p* = 0.028).

**Conclusion:**

irAE was commonly observed in Thai cancer patients treated with ICIs. Most irAEs were low-grade and manageable. Re-occurrence of irAE after re-challenging ICI was not uncommonly observed. Patients who experienced irAEs might have significantly longer OS compared to patients without irAEs. However, OS in this study should be interpreted with caution since it might be affected by various tumor types, treatment settings, dosing schedule, and ICI combinations.

## Background

Cancer immunotherapy, specifically immune checkpoint inhibitors (ICI), are used in monotherapy, combination therapy, or jointly with chemotherapeutic agents and/or targeted therapies. They have become the standard of care for various types of cancers due to significant improvement of clinical and long-term survival outcomes [1].

Use of ICIs can lead to a broad spectrum of autoimmunity-like symptoms – termed immune-related adverse events (irAEs) – that can affect multiple organs, most commonly the endocrine glands, gastrointestinal tract, liver, and skin [2]. Thus, irAEs present a significant challenge in clinical practice, yet the mechanisms underlying irAE development remain unclear. Several potential mechanisms include (i) increasing T-cell activity against common antigens present in both tumors and normal tissue, (ii) increasing levels of pre-existing autoantibodies, (iii) increasing the levels of proinflammatory cytokines, and (iv) enhancing complement-mediated inflammation due to direct binding of an anti-CTLA-4 antibody with CTLA-4 expressed on normal tissue, such as the pituitary gland [2].

The severity of irAEs range from asymptomatic, to mild, to life-threatening [3]. Characteristics and patterns of irAEs may also vary, depending on the ICI’s mechanism of action and the combination used [3]. Moreover, factors predictive of irAEs in ICI-treated cancer patients remain largely unknown [2], though several have been proposed, such as germline genetic factors and host microbiota composition [4–6]. Though variation in incidence and patterns of irAEs in different ethnicities have not been well studied, a sub-group analysis of Asian vs. non-Asian populations in several prospective Phase III studies reported different incidences of specific irAEs between these two populations [7–11]. For instance, a Phase III KeyNote-048 study of pembrolizumab with or without chemotherapy in patients with recurrent/metastatic head and neck squamous cell carcinoma reported that immune-related pneumonitis was more prominent in non-Asians when compared with the Asian population [7]. Moreover, a Phase III CheckMate-025 study of nivolumab monotherapy in advanced renal cell carcinoma reported that the incidence of any-grade endocrine and renal related irAEs was lower, whereas hepatic-related irAE incidence was higher in Japanese subjects when compared to the global population [9]. To date, there is no data of irAE characteristics and outcomes reported in Thai cancer patients treated with ICIs. Therefore, in a large multicenter study, we evaluated the incidence and clinical patterns of irAEs in ICI-treated cancer patients in Thailand to explore factors predictive of immune-related toxicity in high-risk patients, and outcomes of irAE treatment and survival.

## Methods

### Study design

All patients with solid tumors who were treated with any immune checkpoint inhibitors at 3 cancer centers: King Chulalongkorn Memorial, Siriraj, and Ramathibodi hospitals between November 2013 to December 2019 were identified through each center’s database. Available medical records were then retrospectively reviewed using a central database and standardized electronic case record forms. Hematologic malignancy and patients treated with cell or therapeutic vaccine therapy were excluded. Patient baseline characteristics including age and sex, smoking status, Eastern Cooperative Oncology Group (ECOG) performance status, comorbidities of interest, and tumor types were abstracted. Tumor histology, PD-L1 expression, and previous treatments were reviewed. For patients who tested for PD-L1 expression, PD-L1 assay and cut-off for positivity was defined based on standard criteria for each anti-PD1/PD-L1 drugs. Survival outcomes were also collected. Overall survival (OS) was defined as the duration from date of immune-checkpoint inhibitor treatment initiation to death or last follow-up. The survival status of patients was verified and crosschecked with the National Security Death Index. Ethics approval was obtained through either the ethics committee (EC) at each study center. All procedures performed in this study were in accordance with the ethical standards of the institutional research committee and with the 1964 Helsinki declaration and its later amendments or comparable ethical standards.

### Immunotherapy treatment

Eligible patients treated with any immune checkpoint inhibitors in clinical trials, compassionate usage, and clinical practice settings were included in the study. Immune checkpoints inhibitors evaluated in this study included anti-PD1, anti-PD-L1, and anti-CTLA-4. The dose and schedule of each immune checkpoint inhibitor in this study were determined by recommended dose per clinical trial or standard recommended dose in compassionate usage programs or clinical practice. However, for patients who received immune checkpoint inhibitors in a clinical practice setting, the dose and schedule of each drug might be adjusted at the treating physician’s discretion.

### Immune related adverse events

All treatment related toxicities, including irAEs, were graded using the Common Terminology Criteria for Adverse Events (CTCAE) version 4.0 [12]. All irAEs were categorized based on primary organ involvement and maximum grade by CTCAE v.4.0. Characteristics of irAEs including time to onset, duration of irAEs, specific treatments, and outcomes of irAEs were reviewed. irAEs were considered fully resolved when the irAEs return to grade 0 without any treatment-specific medications such as systemic steroids, thyroxine supplements, or anti-thyroid drugs. irAEs were managed and treated according specific guidelines for patients in clinical trials, whereas patients with irAEs outside of clinical trials were treated following standard recommendations and guidelines available at the time of irAE diagnosis at the treating physician’s discretion [13–16].

### Statistical analysis

The study aims to evaluate the incidence of irAEs and their characteristics, treatments, and outcomes in Thai patients who received ICIs as a primary objective. An impact of irAE occurrence on survival was evaluated as a secondary objective. Baseline demographic data was described using descriptive analysis. Categorical variables were presented as number of patients and percentage then Chi-square or Fisher’s exact test was used to compare variables between the irAE and no irAE groups.

Overall survival (OS) was estimated by the Kaplan-Meier method, and the log-rank test was used to compare between irAE and no irAE groups. Univariate Cox regression analysis was performed to identify prognostic factors for OS. Factors with *p*-values < 0.1 from the univariate Cox regression analysis along with irAE, setting of treatment, and ICI use were considered in a multivariate analysis.

To identify factors associated with irAE occurrence, simple logistic regression analysis was used. Then factors with *p*-values less than 0.1 from the simple logistic regression analysis were considered in a multiple logistic regression analysis. All analyses were done on STATA version 15 (Stata Corp., College Station, Texas, USA). A *p*-value < 0.05 was considered statistically significant.

## Results

### Baseline characteristics

A total of 414 eligible patients were identified from the 3 centers. Baseline patient characteristics are listed in Table 1. Overall, irAEs of any grade occurred in 98 of 414 patients (23.7%). Elderly patients (age ≥ 65 years-old) (54.6%), and males (69.3%) made up the majority of ICI-treated individuals in this study. Primary lung cancer (46.6%) and adenocarcinoma (51.2%) were the most common tumor types in the study. Among patients who were tested for PD-L1 expression, 70 of 109 patients tested positive (64%), although the majority of patients in the study were not tested for PD-L1 expression. 167 of 414 patients received prior radiotherapy before ICI treatment was initiated. Only 32 and 15 patients received previous radiation to the lungs and mediastinal areas, respectively, prior to immunotherapy treatment. The majority of patients received prior (neo)adjuvant chemotherapy before initiation of ICI treatment in metastatic setting. irAEs were significantly associated with a prior history of hypothyroidism (*p* < 0.001). Majority of patients (98.4%) had stage 4 disease. There was no statistically significant difference in smoking status, PD-L1 expression, and previous treatment among patients with and without irAEs.

**Table 1:** Patient characteristics

Patient characteristics	All ***N*** = 414	No irAE ***N*** = 316	irAE ***N*** = 98	***p***-value
**Median Age (range)**	63 (17–97)	64 (17–97)	63 (20–91)	
< 65 years-old	226 (54.6)	170 (53.8)	56 (57.1)	0.561
≥ 65 years-old	188 (45.4)	146 (46.2)	42 (82.9)	
**Gender**				0.627
Male	287 (69.3)	221 (69.9)	66 (67.4)	
Female	127 (30.7)	95 (30.1)	32 (32.6)	
**Smoking status**				0.989
Never	142 (34.3)	108 (34.2)	34 (34.7)	
Active or ex-smoker	142 (34.3)	109 (34.5)	33 (33.7)	
Unknown	130 (31.4)	99 (31.3)	31 (31.7)	
**ECOG status**				0.431
0–1	394 (95.2)	299 (94.6)	95 (96.9)	
≥ 2	20 (4.8)	17 (5.4)	3 (3.1)	
**Comorbidity of Interest**				
Hypothyroidism	15 (3.6)	6 (1.9)	9 (9.2)	0.001
Hyperthyroidism	5 (1.2)	4 (1.3)	1 (1.0)	1.000
DM	68 (16.4)	53 (16.8)	15 (15.3)	0.732
Chronic HBV	28 (6.8)	24 (7.6)	4 (4.1)	0.355
Chronic HCV	9 (2.2)	8 (2.5)	1 (1.0)	0.692
**Primary Tumor types**				0.031
Lung cancer	193 (46.6)	156 (49.4)	37 (37.8)	
HCC^a^	42 (10.1)	30 (9.5)	12 (12.2)	
Bladder cancer/TCC^a^	40 (9.7)	30 (9.5)	10 (10.2)	
Melanoma	33 (8.0)	21 (6.7)	12 (12.2)	
HNSCC^a^	23 (5.6)	19 (6.0)	4 (4.1)	
RCC^a^	20 (4.8)	16 (5.1)	4 (4.1)	
Nasopharyngeal carcinoma	19 (4.6)	12 (3.8)	7 (7.2)	
Esophageal cancer	8 (1.9)	6 (1.9)	2 (2.0)	
Colorectal cancer	7 (1.7)	4 (1.3)	3 (3.1)	
Breast cancer	6 (1.4)	6 (1.9)	0	
Pancreatic cancer	4 (1.0)	1 (0.3)	3(3.1)	
Stomach/EGJ^a^ cancer	3 (0.7)	1 (0.3)	2 (2.0)	
Cholangiocarcinoma	2 (0.5)	1 (0.3)	1 (1.0)	
Others	14 (3.4)	13 (3.0)	1 (1.0)	
**Histology**				0.122
Adenocarcinoma	212 (51.2)	163 (51.6)	49 (50.0)	
Squamous Cell	79 (19.1)	58 (18.3)	21 (21.4)	
Transitional cell carcinoma	41 (9.9)	31 (9.8)	10 (10.2)	
Melanoma	36 (8.7)	23 (7.3)	13 (13.3)	
Clear cell	15 (3.6)	12 (3.8)	3 (3.1)	
Others	31 (7.5)	29 (9.2)	2 (2.0)	
**PD-L1 status**				0.065
Negative	39 (9.4)	29 (9.2)	10 (10.2)	
Positive	70 (16.9)	61 (19.3)	9 (9.2)	
Unknown	305 (73.7)	226 (71.5)	79 (80.6)	
**Previous Treatment**				
Chemotherapy	250 (60.4)	198 (62.7)	52 (53.1)	0.090
Radiotherapy	167 (40.3)	128 (40.5)	39 (39.8)	0.900
Site of previous Radiotherapy				
Lung	32 (7.7)	24 (7.6)	8 (8.2)	0.854
Mediastinum	15 (3.6)	10 (3.2)	5 (5.1)	0.370
Neck/thyroid	24 (5.8)	17 (5.4)	7 (7.1)	0.514

^a^*TCC* transitional cell carcinoma, *HCC* hepatocellular carcinoma, *HNSCC* head and neck squamous cell carcinoma, *RCC* renal cell carcinoma

### Immunotherapy treatment

Most ICIs in this study (51.7%) were delivered in a first-line palliative setting (Table 2). Only 6 patients (1.4%) received adjuvant ICIs, and all of them were treated in a clinical trial setting. In patients who received monotherapy ICI, 25 of 322 patients (7.8%) were treated with anti-CTLA4, whereas 297 patients (92.2%) received anti-PD1/PD-L1. Patients who received ICIs in clinical trial settings had significantly more frequent irAEs (60.2%) than those in compassionate use programs (5.1%) and those in clinical practice (34.7%); *p* < 0.001. Nineteen patients received combined immunotherapy including anti-PD-1/PD-L1 and anti-CTLA4. Eleven of these 19 patients (57.9%) developed irAEs, whereas 72 of 322 patients (22.4%) treated with single agent immunotherapy had irAEs (*p* = 0.004).

**Table 2:** Immunotherapy Treatment

Treatment	All ***N*** = 414	No irAE ***N*** = 316	irAE ***N*** = 98	***p***-value
**Settings**				< 0.001
Clinical trial	161 (38.9)	102 (32.3)	59 (60.2)	
Compassionate program	31 (7.5)	26 (8.2)	5 (5.1)	
Clinical practice	222 (53.6)	188 (59.5)	34 (34.7)	
**Treatment setting**				0.074
Adjuvant	6 (1.4)	5 (1.6)	1 (1.0)	
1st line metastasis	214 (51.7)	152 (48.1)	62 (63.3)	
2nd line metastasis	113 (27.3)	92 (29.1)	21 (21.4)	
≥ 3rd line metastasis	81 (19.6)	67 (21.2)	14 (14.3)	
**Immunotherapy combination**				0.004
Single agent	322 (77.8)	250 (79.1)	72 (73.5)	
Combination ICI + ICI	19 (4.6)	8 (2.5)	11 (11.2)	
Combination ICI + chemotherapy	62 (15.0)	50 (15.8)	12 (12.2)	
Combination ICI + others	11 (2.7)	8 (2.5)	3 (3.1)	

*ICI* immune checkpoint inhibitor, *irAE* immune related adverse events

### Immune-related adverse events, their treatments and outcomes

3The overall incidence of all grade irAEs in this study was 98 of 414 patients (23.7%) (Table 3). The most frequently occurring all grade irAEs were hypothyroidism (7.5%), hepatitis (6.5%), and rash (4.8%). The incidence of severe adverse events (grades 3–5) was 5.6% of all patients. Six of four hundred fourteen patients (1.4%) had all grade immune-related pneumonitis, whereas 3 patients had severe pneumonitis (grade 3–5) and were treated with high dose systemic steroids intravenously. One patient with grade 1 pneumonitis did not receive systemic steroids and symptoms resolved after holding the ICI. Another 2 patients with grade 2 pneumonitis were treated with oral prednisolone until recovery. No patient was treated with infliximab. Hepatitis was reported in 27 of 414 patients (6.5%), of which 22 of 27 patients had grade 1–2 hepatitis and did not require systemic steroids. Only 5 patients with grade 3–4 hepatitis were treated with systemic steroids. All grade hepatitis was completely resolved in 23 of 27 patients (85.2%) at the time of data cut-off.

**Table 3:** Incidence, severity, treatment and outcomes of patients who had irAEs

irAEs	Grade (***n*** = 414)			Median onset,days (range)	Median Time to Resolution, days (range)	irAE specific Treatment (n, %)	Termination of ICI due to irAE (n, %)	irAE Recovery (n, %)	ICI rechallenge (n, %)	Recurrence of irAE after rechallenge (n, %)
	All	Gr 1–2	Gr 3–5							
**Overall**	98 (23.7)	75 (18.1)	23 (5.6)	63 (1–526)	51 (1–951)	56/98 (57.1)	22/98 (22.5)	84/98 (85.7)	68/98 (69.4)	11/68 (11.2)
**Endocrine**										
Hypothyroid	31 (7.5)	31 (7.5)	–	112 (19–574)	168 (11–804)	25/31 (80.7)	5/31 (16.1)	25/31 (80.7)	21/31 (67.7)	1/21 (4.8)
Hyperthyroid	11 (2.7)	11 (2.7)	–	47 (15–203)	35 (12–751)	6/11 (54.6)	0	10/11 (90.9)	8/11 (72.7)	0/8
Hypophysitis	5 (1.2)	4 (1.0)	1 (0.2)	178 (1–348)	208 (54–807)	5/5 (100)	1/5 (20.0)	5/5 (100)	4/5 (80.0)	0/4
**GI**										
Colitis	1 (< 0.01)	–	1 (< 0.01)	475	181	1	0	1	1	0
Hepatitis	27 (6.5)	22 (5.3)	5 (1.2)	42 (7–400)	42 (7–632)	5/27 (18.5)	8/27 (29.6)	23/27 (85.2)	17/27 (63.0)	3/17 (17.7)
Pancreatitis	11 (2.7)	9 (2.2)	2 (0.5)	30 (14–254)	84 (1–707)	1/11 (9.1)	1/11 (9.1)	9/11 (81.8)	10/11 (90.9)	4/10 (40.0)
**Lung**										
Pneumonitis	6 (1.4)	3 (0.7)	3 (0.7)	34 (23–139)	18.5 (8–33)	5/6 (83.3)	2/6 (33.3)	6/6 (100)	3/6 (50.0)	0/3
**Skin**										
Rash	20 (4.8)	17 (4.1)	3 (0.7)	55.5 (3–420)	34 (7–253)	14/20 (70.0)	4/20 (20.0)	18/20 (90.0)	17/20 (85.0)	4/17 (23.5)
Vitiligo	1 (< 0.01)	1 (< 0.01)	–	70	951	1	0	0	1	1
**Others**										
Others	8 (2.0)	4 (1.0)	4 (1.0)	181.5 (2–505)	31.5 (20–86)	4/8 (50.0)	2/8 (25.0)	7/8 (87.5)	4/8 (50.0)	0/4

*Gr* grade, *irAE* immune related adverse events, *ICI* immune checkpoint inhibitor, *GI* gastrointestinal adverse events, *n* number of patients

Median time to onset of overall irAEs was 63 days (range 1–526). Pancreatitis and pneumonitis had the earliest irAE onset of 30 and 34 days, respectively. Hypothyroidism and hypophysitis had the longest median time to resolution of all irAEs (186 and 208 days, respectively). Twenty-two of ninety-eight patients with irAEs had a permanent discontinuation of ICIs due to toxicity. The majority of irAEs in this study completely resolved (85.7%), either with irAE-specific treatments or discontinuation of immunotherapy. ICIs were rechallenged in 68 of 98 patients with irAE (69.4%) when the irAEs recovered to grade 1 or less. Subsequently, 11 of 68 patients with irAEs (11.2%) had a recurrence of irAEs after ICI rechallenge. Pancreatitis (40%), skin rash (23.5%), and hepatitis (17.7%) were the most common manifestations of irAE recurrence. No irAE-related death (grade 5) was observed in this study. The median duration of follow-up was 9.6 months. Patients with irAEs had significant longer median duration of follow-up when compared with patients without irAEs (13.4 vs 8.1 months; *p* = 0.005).

Univariate and multivariate analyses for irAE occurrence Odds Ratio (OR) are depicted in Table 4. In the multivariate analysis for occurrence of irAE OR, history of hypothyroidism (OR = 7.21, 95% CI 2.38–21.81; *p* < 0.001), immunotherapy use in clinical trial settings (OR = 3.43, 95% CI 2.10–5.60; *p* < 0.001), and combination of immunotherapy/ immunotherapy (OR = 5.30, 95% CI 1.96–14.33; *p* < 0.001) were independent factors predicting occurrence of irAE. In patients with a previous history of hypothyroidism who were on levothyroxine supplementation without recent levothyroxine dose adjustment, 10 of 15 patients (67%) developed irAEs after initiation of ICI. Five of 10 patients had worsening hypothyroidism. One patient developed hyperthyroidism, for which levothyroxine supplementation was discontinued. Another 3 patients had hepatitis, pneumonitis, and skin rash, respectively, without worsening of their previous hypothyroidism.

**Table 4:** Univariate and multivariate logistic regression analyses for Odd Ratio for occurrence of irAE

Factors	N	Event	Univariate		Multivariate	
			OR (95%CI)	***p***-value	OR (95%CI)	***p***-value
**Age**						
< 65	226	56	1.15 (0.72, 1.81)	0.561	–	–
≥ 65	188	42	1			
**Sex**						
Male	287	66	0.89 (0.55, 1.44)	0.627	–	–
Female	127	32	1			
**Smoking history**						
Never	142	34	1.04 (0.60, 1.80)	0.889	–	–
Ever	142	33	1			
Unknown	130	31	1.03 (0.59, 1.81)	0.906	–	–
**History of hypothyroidism**						
No	399	89	1			
Yes	15	9	5.22 (1.81, 15.07)	**0.002**	7.21 (2.38, 21.81)	**< 0.001**
**Histology Subtype**						
Squamous	79	21	1.21 (0.69, 2.12)	0.499	–	–
Non-Squamous	335	77	1			
**Treatment Setting**						
Clinical trial	161	53	3.17 (1.99, 5.07)	**< 0.001**	3.43 (2.10, 5.60)	**< 0.001**
Non-clinical trial	253	39	1		1	
**Immunotherapy combination**						
Monotherapy	322	72	1		1	
ICI + ICI	19	11	4.77 (1.85, 12.32)	**0.001**	5.30 (1.96, 14.33)	**0.001**
ICI + Chemotherapy/others	73	15	0.90 (0.34, 5.04)	0.736	0.82 (0.42, 1.58)	0.550

*N* number of patients, *OR* odd ratio, *ICI* immune checkpoint inhibitor

### Overall survival

**Fig. 1:**
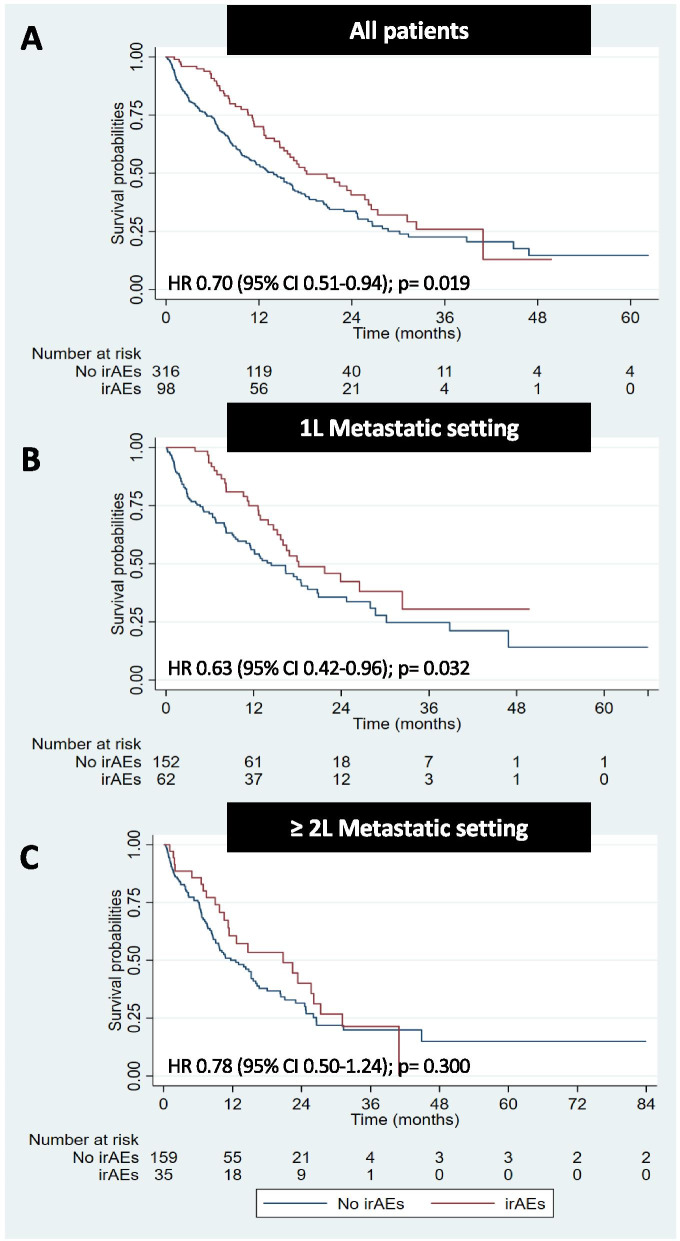
Overall survival (OS) of all solid tumor patients (**A**), solid tumor patients who received immunotherapy in 1 L (**B**), and ≥ 2 L (**C**) metastatic settings with and without irAE

**Fig. 2:**
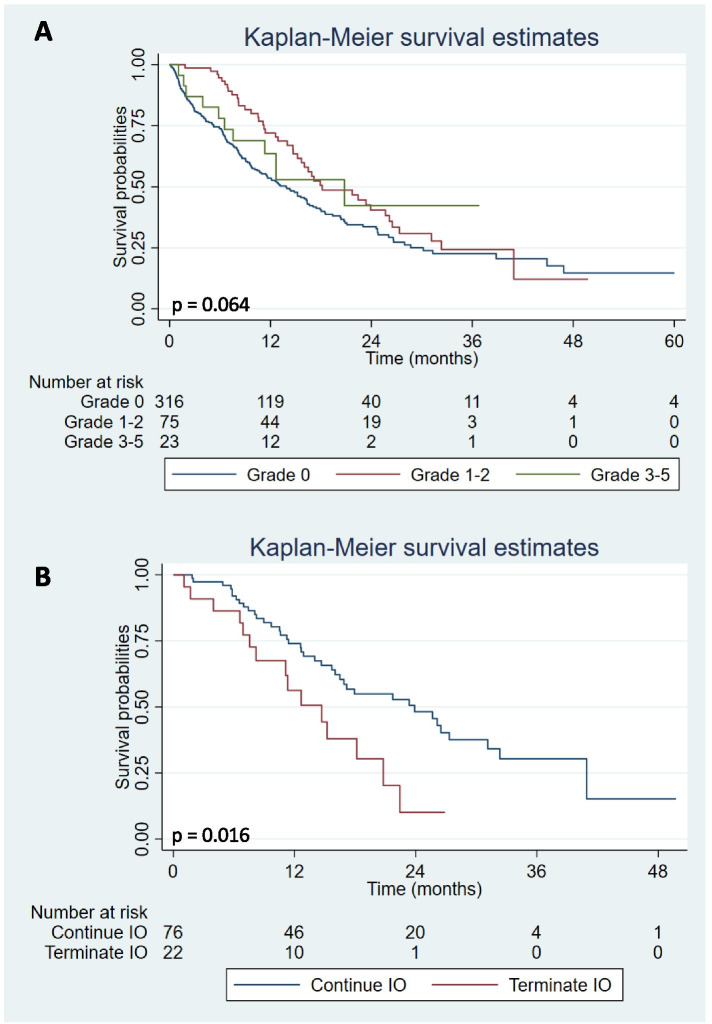
OS of solid tumor patients by the severity of irAEs using the CTCAE version 4.0 (**A**), OS of solid tumor patients with irAEs who continued and discontinued ICIs after the first irAE occurrence (**B**)

The median OS of the overall cohort was 15.2 months. Overall, patients with irAEs had significantly longer OS when compared to patients without irAEs (18.2 vs 13.9 months; *p* = 0.019) (Fig. 1A). Univariate and multivariate analyses for OS are shown in Table 5, which demonstrate that irAE occurrence was an independent prognostic factor for OS (HR 0.70, 95% CI 0.51–0.96; *p* = 0.028). In the patients who received ICIs as a first line treatment for metastatic disease, the median OS of patients who had irAEs was 18.2 months, compared with 14.4 months for patients without irAEs (HR 0.63, 95% CI 0.42–0.96; *p* = 0.032) (Fig. 1B). However, there was no statistically significant difference among patients who received ICIs as a second or later line treatment for metastatic disease (20.8 vs 12.6 months; *p* = 0.300), and severity of irAE grade by the CTCAE version 4.0 (*p* = 0.064) (Figs. 1, and 2C and A). However, solid tumor patients with irAEs who continued ICIs after the first occurrence of irAEs had significantly longer OS when compared to patients who ICIs were discontinued due to irAEs (23.9 vs. 14.7 months; *p* = 0.013) (Fig. 2B).

**Table 5:** Univariate and multivariate Cox regression analysis for overall survival (OS)

Factors	N	OS (months)	Univaritate		Multivariate	
			HR (95%CI)	***p***-value	HR (95%CI)	***p***-value
**Age**						
< 65	226	15.2	1.01 (0.78, 1.30)	0.964	–	–
≥ 65	188	15.7	1			
**Sex**						
Male	287	16.0	0.93 (0.71, 1.21)	0.576	–	–
Female	127	15.2	1			
**Smoking history**						
Never	142	15.2	0.94 (0.70, 1.27)	0.686	–	–
Ever	142	13.9	1			
Unknown	130	15.2	0.92 (0.66, 1.28)	0.610	–	–
**Histology Subtype**						
Squamous	79	15.2	1.16 (0.86, 1.58)	0.335	–	–
Non-Squamous	335	15.2	1			
**Treatment Setting**						
Clinical trial	161	16.4	0.84 (0.65, 1.09)	0.192	0.90 (0.69, 1.18)	0.450
Non-clinical trial	253	14.7	1		1	
**Immunotherapy combination**						
Monotherapy	322	15.2	1		1	
ICI + ICI	19	15.2	1.07 (0.60, 1.92)	0.819	1.20 (0.67, 2.18)	0.539
ICI + Chemotherapy/other	73	16.0	0.89 (0.61, 1.29)	0.530	0.88 (0.61, 1.28)	0.512
**irAEs**						
Yes	98	18.2	0.70 (0.51, 0.94)	**0.020**	0.70 (0.51, 0.96)	**0.028**
No	316	13.9	1		1	

*N* number of patients, *OS* overall survival, *ICI* immune checkpoint inhibitor, *irAE* immune related adverse events

## Discussion

We report the largest multicenter study that describes the characteristics and outcomes of solid tumor patients treated with immunotherapy in Thailand. The overall incidence of irAEs in Thailand was consistent with the literature [3, 7–11, 17, 18], where more than 90% of our patients was treated with anti-PD1/PD-L1 monotherapy. However, the incidence of each specific organ system–based irAEs might be slightly different due to various types of ICIs (or its combinations) used, different dosages, as well as unique patient populations. Several studies suggest that the tumor microenvironment and gut microbiota may also influence the risk and patterns of irAEs [4, 5]. Since most patients in our study received anti-PD1/PD-L1 monotherapy, irAEs that are most commonly associated with anti-CTLA4 therapy or its combinations thereof, such as colitis, and diarrhea were rare [3]. Endocrinopathies of all grades, especially hypothyroidism, were most commonly reported and the incidence was consistent with previous reports from global and Asian populations [3, 7–11]. The clinical manifestation of thyroid disorders in our patients mostly consisted of early onset thyrotoxicosis or asymptomatic subclinical hypothyroidism, with subsequent transitioning to hypothyroidism that required long-term levothyroxine supplementation. This pattern was consistent with a previous report of immune-related thyroiditis with immune checkpoint inhibitors [19, 20]. Similarly, life-threatening irAEs such as immune-related pneumonitis were uncommon with an incidence of 1.4% in our study, which was comparable with the rate reported in the literature [3, 7–11, 17, 18, 21].

In our study, pancreatitis and pneumonitis had the earliest onset of approximately 4 weeks, while hypothyroidism had the latest onset of almost 12 weeks after initiation of ICIs. In a pooled analysis of patients with advanced melanoma who received nivolumab monotherapy, patterns and characteristics of selected treatment-related AEs were described [18]. Skin and GI toxicities had the shortest median time to onset of 5.0 and 7.3 months, respectively [18]. Endocrinopathy occurred at 10.4 weeks, whereas pulmonary toxicity was observed at 8.9 weeks after starting nivolumab [16]. On the other hand, patients treated with ipilimumab had skin, and GI-related AEs after 2–3 weeks, and 6 to 7 weeks, respectively [22]. Endocrinopathy occurred after an average of 9 weeks of ipilimumab treatment [22]. In our study, skin rash had the shortest median time to resolution of almost 5 weeks, while Weber, et al. reported a median time to resolution of 18 weeks for skin toxicity in advanced melanoma patients treated with nivolumab [18]. In this study, we reported that within the 69% of patients who were re-challenged with immunotherapy after their irAEs resolved to grade 1 or less, 11% experienced irAE recurrence. To our knowledge, there are limited data on immunotherapy rechallenge and irAE reoccurrence in the literature. In cases where the ICI continues to benefit patients with non-life-threatening irAEs, ICI rechallenge when appropriate might be possible since the recurrence rate of irAEs was acceptable.

In this retrospective, patients involving in multicenter clinical studies, immunotherapy combination and history of hypothyroidism were independent predictors of irAE occurrence, which is consistent with the literature. Patients in a prospective clinical trial would have more aggressive monitoring of irAE leading to more accurate toxicity reports when compared to those in usual clinical practice or in compassionate usage program. The combination of two ICIs has been known to increase irAE risk when compared to single agents [17, 21]. Previous retrospective studies reported that elevated levels of baseline thyroid stimulating hormone (TSH), and pre-existing anti-thyroid antibodies were significantly associated with immune-related thyroid dysfunction in patients treated with ICIs [20, 23].

Our study demonstrated that patients with irAEs had significantly longer OS in both univariate and multivariate analyses. However, patients who developed irAEs had longer follow-up duration when compared with patients who did not. Therefore, survivorship bias should be considered to interpret the relationship between irAEs occurrence and favorable survival. Although the association of irAE occurrence and survival of cancer patients treated with ICIs remains controversial, most studies suggest that irAE occurrence was associated with ICI efficacy [18, 20, 24–27]. A meta-analysis of 30 studies demonstrated a significant association between irAE occurrence and better outcome of ICI-treated solid tumor patients, in particular for anti-PD-1 or PD-L1 inhibitors [24].

Our study had several limitations. Though almost 40% of patients in our study received ICIs in a prospective clinical trial setting, incidences of low grade asymptomatic irAEs might be overlooked and limited in the retrospective part of the study. Survival outcomes might be affected by various tumor types, treatment settings (adjuvant, 1st, and later line metastatic), dosing schedule, and ICI combinations. Moreover, the study might have relatively short follow-up time although almost half of patients in the study received ICIs as the 2nd or later line treatment for metastatic disease. Therefore, survival outcomes in this study should be interpreted with caution.

Since all commercially available ICIs are expensive and not reimbursable by major insurance policies in Thailand, the majority of Thai patients have limited access to these drugs. Though this is the largest multicenter study describing characteristics and outcomes of irAE treatment in Thailand, the study is limited by data collection which occurred in both a prospective (for clinical trials) and retrospective manner. In addition, multiple ICIs and their combinations were included in this study although the majority of patients were treated with anti-PD1 or PD-L1 inhibitor monotherapy. The study mostly describes patterns and outcomes of irAEs in solid tumor patients treated with ICIs in Thailand. The association of predictive factors of irAE occurrence and survival should be cautiously interpreted.

## Conclusion

irAEs were commonly observed in Thai cancer patients treated with ICIs. Most irAEs were low-grade and manageable following current practice guidelines. Recurrence of irAEs after ICI rechallenge was not uncommon. Patients who experienced irAEs might have significantly longer OS compared to patients without irAEs. However, OS in this study should be interpreted with caution since it might be affected by various tumor types, treatment settings, dosing schedule, and ICI combinations.

## Data Availability

The datasets used and/or analysed during the current study available from the corresponding author on reasonable request.

## Abbreviations

CTLA-4: Cytotoxic T-lymphocyte antigen 4
ECOG: Eastern Cooperative Oncology Group
HCC: Hepatocellular carcinoma
HNSCC: Head and neck squamous cell carcinoma
ICI: Immune checkpoint inhibitors
irAE: Immune-related adverse event
PD-L1: Program death ligand-1
RCC: Renal cell carcinoma
TCC: Transitional cell carcinoma
OR: Odd ratio
OS: Overall survival
